# Post-Operative Outcomes of Circular External Fixation in the Definitive Treatment of Tibial Plafond Fractures: A Systematic Review

**DOI:** 10.7759/cureus.24204

**Published:** 2022-04-17

**Authors:** Peter I Legg, Khalid Malik-Tabassum, Yasser H Ibrahim, Baljinder S Dhinsa

**Affiliations:** 1 Trauma and Orthopaedics, William Harvey Hospital, Ashford, GBR; 2 Trauma and Orthopaedics, Brighton & Sussex University Hospitals, Brighton, GBR; 3 Trauma and Orthopaedics, Conquest Hospital, Hastings, GBR

**Keywords:** distal tibia fracture, external fixation, circular external fixator, tibial plafond, pilon

## Abstract

Tibial plafond fractures (TPFs) are uncommon but potentially devastating injuries to the ankle. Operative treatments include internal and external fixation modalities. This article provides a systematic review of the clinical and functional outcomes of TPFs treated specifically with circular external fixation (CEF). A literature search of medical databases from inception to 13th November 2020 was performed. Original studies written in the English language reporting clinical, radiological, and functional outcome data of TPF treated with CEF were included. Patient demographics, fracture classification, open fractures, post-operative complications, clinical outcomes, radiological outcomes, and functional outcomes were collected. Quality and risk of bias were assessed using standardised scoring tools.In total, 16 studies were included. One prospective randomised study was identified. Collated data of 303 patients were analysed. The mean time to union was 21 weeks. Malunion occurred in 12.4%. The rate of deep infection was 4.8%, but no amputations were recorded. The risk of minor soft tissue infection (including pin-site infections) was 54%. Almost two-thirds achieved good-to-anatomic reduction radiologically. Approximately one-third reported excellent functional outcome scores. The quality of the studies was deemed satisfactory. A moderate risk of bias was acknowledged. This systemic review provides a summary of outcome data regarding CEF as a treatment for TPF. It highlights CEF as an acceptable treatment option with comparable results to that of internal fixation. Further higher-quality evidence is advised.

## Introduction and background

Pilon or tibial plafond fractures (TPFs) constitute 7-10% of all tibial fractures and are typically high-energy injuries, with a combination of rotational and axial forces resulting in impaction of the talar dome into the distal tibial articular surface [1,2]. Aptly, the term “pilon” arises from the French word for pestle, the round-ended tool used to crush or pound spices [3,4]. The action of the talus crushing into the tibial plafond (a French portmanteau: plat = flat and fond = base) results in these intra-articular fractures, often exhibiting a combination of articular impaction, metaphyseal comminution, and significant soft tissue injury. Two classifications are commonly used to describe bony injury pattern of TPF: The Arbeitsgemeinschaft für Osteosynthesefragen/Orthopedic Trauma Association (AO/OTA) and Ruedi-Allgower (RA type I, II, III). The aim of treatment is to restore articular congruency, restore tibial length, alignment and rotation, provide stability to allow healing, and commence early range of movement. The complexity of TPF, including the bony and soft tissue injury, presents a significant challenge to orthopaedic surgeons, and patient-related outcomes are both variable and unpredictable [5]. A key principle in managing TPF remains meticulous care of the soft tissue envelope [6]. Early open reduction internal fixation (ORIF) has been associated with high complication rates [7-9]. This has led to increased use of staged ORIF and external fixation (EF). The theoretical advantage of EF over ORIF is in achieving indirect reduction while reducing insult to the already compromised soft tissue envelope [10,11]. A combination of limited ORIF (LIF) and EF has been used to further aid reduction if necessary [5]. EF constructs can take many forms. However, circular-frame external fixation (CEF) (i.e., Ilizarov and Taylor Spatial Frames, TSF) have been shown to be biomechanically superior, improve indirect reduction and deformity correction, and allow earlier mobilization when compared to other forms of EF [1,12-15]. While staged ORIF continues to be the “standard of care,” CEF tends to be reserved for patients with more severe soft tissue injury [16]. The aim of this systematic review is to report the post-operative complications and functional outcomes in TPFs undergoing definitive treatment with CEF.

## Review

Materials and methods

**Figure 1:**
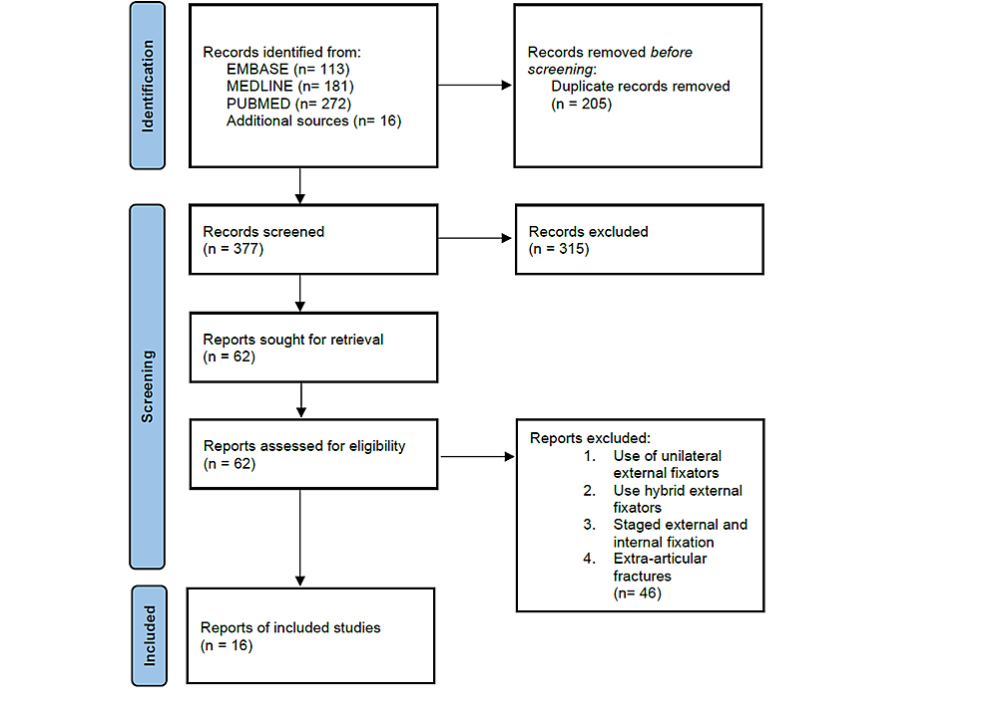
PRISMA diagram summarising the data collection process. PRISMA: Preferred Reporting Items for Systematic Reviews and Meta-Analyses

This systematic review was performed in accordance with the Preferred Reporting Items for Systematic Reviews and Meta-Analyses (PRISMA) guidelines (Figure 1) [17]. Two authors (PL, KM) independently searched MEDLINE, EMBASE, PubMed, and Cochrane Library electronic databases from their inception to the date of the final search (13th November 2020). Boolean operators were used in addition to the search terms: “tibial pilon fracture,” “tibial plafond fracture” AND “circular frame,” “circular external fixat*,” Ilizarov frame,” “taylor spatial frame OR TSF,” “hexapod” and “ring fixator.”

Original articles published in the English language were included. Studies reporting clinical or functional outcomes of CEF for definitive treatment of TPF (including open and closed fractures) either isolated or as part of polytrauma were included. Follow-up periods of less than 12 months were excluded. Patients under the age of 18 years, case reports, animal, cadaveric, and biomechanical studies, conference papers, abstracts, and review articles were also excluded.

The primary outcome measures were bone-healing complications (non-union, mal-union), superficial infection (pin-site infections and superficial wound infections not requiring surgical intervention), deep infection (soft tissue or osteomyelitis requiring surgical intervention, including debridement and revision or removal of implants), and limb amputation. The secondary outcome measures included patient-reported outcome measures (PROMS) and radiological outcomes.

The methodological quality of the studies was assessed using the Methodological Index for Non-Randomized Studies (MINORS) score [18]. Bias risk was assessed using the Risk Of Bias In Non-Randomised Studies - of Interventions (ROBINS-I) score [19]. Level of evidence was determined based on the classification by Wright et al. [20].

Results

The literature search identified 582 studies. After removal of duplicates and clear exclusions, the references of the 62 eligible articles were also screened to identify any additional relevant articles. A total of 16 articles met the inclusion criteria for analysis (Table 1).

**Table 1:** Study characteristics by article. NR denotes data not reported. AO = AO/OTA classification; RA = Ruedi-Allgower classification I, II, III = RA type; B/C = AO/OTA type; AO = Arbeitsgemeinschaft für Osteosynthesefragen; OTA = Orthopedic Trauma Association

Author	Publication year	cohort size, n	Frame duration, weeks	Time to union, weeks	Non-union	Mal-union	Revision of frame	Minor soft tissue infection	Major soft tissue infection	Osteomyelitis	Nerve injury	Secondary tibiotalar arthrodesis
McDonald et al. [21]	1996	13	11	NR	2	0	0	9	0	0	0	1
Okcu and Aktuglu [15]	2004	24	16.4	NR	0	5	NR	NR	0	0	NR	0
Kapukaya et al. [22]	2005	14	15	NR	0	1	0	17	0	0	0	0
Harris et al. [16]	2006	16	18	NR	1	1	NR	2	1	0	0	1
Vidyadhara and Rao [23]	2006	21	26.6	26.6	0	1	3	7	1	0	0	NR
Bacon et al. [24]	2008	13	NR	24.5	4	3	0	4	1	3	1	1
Lovisetti et al. [25]	2009	30	21.4	21.4	1	2	1	5	1	0	NR	1
Kholeif et al. [26]	2009	15	15.9	NR	0	1	NR	10	0	0	0	NR
Kapoor et al. [27]	2010	17	17	15.8	0	4	1	9	0	0	0	0
Ramos et al. [28]	2013	18	15	NR	1	4	1	15	11	0	0	1
Osman et al. [29]	2017	30	22	NR	0	4	3	10	0	0	0	1
Imren et al. [30]	2017	20	NR	22.1	0	NR	NR	13	1	0	NR	NR
Patra et al. [31]	2017	21	15.5	13.1	0	3	1	19	0	0	0	NR
Sahin et al. [32]	2017	14	NR	26	0	2	0	5	0	0	0	0
Rayan et al. [33]	2018	20	NR	24.5	NR	2	NR	NR	NR	NR	NR	NR
Pirwani et al. [34]	2018	17	18	14.6	0	NR	0	14	8	0	0	0

Table 1 illustrates the characteristics of the 16 studies. The majority of studies were evidence level III or IV; only one level II (prospective, randomised trial) study was identified. Of 303 patients, 70 (23.1%) were open fractures, reporting 202 males, 62 females, and 39 unspecified. Kapoor et al. reported one death post-operatively from polytrauma; therefore, follow-up data were excluded for this individual [27]. The mean age was 41.1 ± 8.0 (standard deviation) years. The mean follow-up time was 35.3 ± 13.9 months.

Primary outcome measures

The mean time in CEF was 17.6 ± 3.9 weeks. The mean time to union was 21.0 ± 4.9 weeks. The non-union rate was 3.2%. The malunion rate was 12.4% (Table 2).

**Table 2:** Primary outcome measures.

Author	Publication year	cohort size, n	Frame duration, weeks	Time to union, weeks	Non-union	Mal-union	Revision of frame	Minor soft tissue infection	Major soft tissue infection	Osteomyelitis	Nerve injury	Secondary tibiotalar arthrodesis
McDonald et al. [21]	1996	13	11	NR	2	0	0	9	0	0	0	1
Okcu and Aktuglu [15]	2004	24	16.4	NR	0	5	NR	NR	0	0	NR	0
Kapukaya et al. [22]	2005	14	15	NR	0	1	0	17	0	0	0	0
Harris et al. [16]	2006	16	18	NR	1	1	NR	2	1	0	0	1
Vidyadhara and Rao [23]	2006	21	26.6	26.6	0	1	3	7	1	0	0	NR
Bacon et al. [24]	2008	13	NR	24.5	4	3	0	4	1	3	1	1
Lovisetti et al. [25]	2009	30	21.4	21.4	1	2	1	5	1	0	NR	1
Kholeif et al. [26]	2009	15	15.9	NR	0	1	NR	10	0	0	0	NR
Kapoor et al. [27]	2010	17	17	15.8	0	4	1	9	0	0	0	0
Ramos et al. [28]	2013	18	15	NR	1	4	1	15	11	0	0	1
Osman et al. [29]	2017	30	22	NR	0	4	3	10	0	0	0	1
Imren et al. [30]	2017	20	NR	22.1	0	NR	NR	13	1	0	NR	NR
Patra et al. [31]	2017	21	15.5	13.1	0	3	1	19	0	0	0	NR
Sahin et al. [32]	2017	14	NR	26	0	2	0	5	0	0	0	0
Rayan et al. [33]	2018	20	NR	24.5	NR	2	NR	NR	NR	NR	NR	NR
Pirwani et al. [34]	2018	17	18	14.6	0	NR	0	14	8	0	0	0

The overall complication rate was 11.3% (225/1,997 reported events). A total of 225 complications were reported. There were 139/245 (56.7%) minor soft tissue infections managed by antibiotics and dressing. The rate of major soft tissue infection or osteomyelitis was 4.8%.

Pin-site infections and superficial wound infections are commonplace in both CEF and fixation of TPF [35]. While overall complication rate is important in both decision-making as a surgeon and providing informed consent to patients, we acknowledge that the inclusion of simple pin-site infections influences the overall complication rate heavily.

Therefore, we define the “serious” infective complication rate by excluding these minor soft tissue infections, resulting in a rate of 4.9% (86/1,738 reported events).

Overall, 10/208 (4.8%) required a return to the theatre for frame adjustment or revision. In total, 6/206 (2.9%) secondary tibiotalar arthrodeses occurred following CEF. No amputations were reported within the follow-up period. The rate of nerve injury was 1/209 (0.5%). Only Bacon et al. documented a nerve injury but did not include any detail regarding this event [24].

Secondary outcome measures

A total of 10 articles reported objective range of motion in plantar- and dorsi-flexion at the final follow (Table 3). Pooled mean dorsiflexion was 11.8 ± 2.4 degrees, and the mean plantar flexion was 24.8 ± 5.2 degrees.

**Table 3:** Range of motion at the final follow-up.

	Cohort, n	Dorsiflexion, degrees	Plantarflexion, degrees
Ramos et al. [28]	18	17.0	19.0
Kapoor et al. [27]	17	9.9	30.9
Osman et al. [29]	30	10.0	22.5
Kapukaya et al. [22]	14	10.9	20.4
Vidyadhara and Rao [23]	21	10.0	20.0
Kholeif et al. [26]	15	11.2	19.5
Patra et al. [31]	21	10.0	31.2
McDonald et al. [21]	13	12.0	25.0
Okcu and Aktuglu [15]	24	11.3	33.5
Sahin et al. [32]	14	15.5	26.1

In total, 11 articles reported standardised functional outcome scores using two standardised scoring systems, of which six provided numerical data (Table 4). Four articles used the Modified Mazur Ankle score (MMAS). Seven used the AO Foot and Ankle score (AOFAS), of which four articles reported mean AOFAS scores, and three reported stratified scores. Imren et al. reported mean AOFAS at one-, two- and three-year interals [30]. Interestingly, the mean score continued to decline from 86.6 to 82.1 to 79.7, respectively. Imren et al., Rayan et al., Patra et al., and Sahin et al. all reported one-year mean AOFAS scores of 86.6, 86.7, 76.3, and 80.4, respectively [30,31,33]. Kapoor et al. reported a mean MMAS score of 79.8 from 16 patients [27]. Of the 113 pooled stratified scores from both systems (Table 4), functional outcome scores were 32.7% excellent, 35.4% good, 21.2% fair, and 10.6% poor. Where only average scores were reported: pooled average scores were AOFAS 80.8 ± 3.8 (n = 75) and MMAS 83.2 ± 3.4 (n = 40). Harris et al. reported Musculoskeletal Functional Assessment scores and Foot Function Index scores with a mean of 34 and 0.4, respectively, but the time of assessment was not stated [16]. Ramos et al. reported Visual Analogue Scores and EQ5D scores with a mean of 18.9 and 0.69, respectively. However, the period of scoring ranged between one and five years [28].

**Table 4:** Functional outcome scores. MMAS (Modified Mazur Ankle Score) score stratification: Excellent >92, Good 87-91, Fair 65-86, Poor <65. AOFAS (American Orthopedic Foot and Ankle Society) score stratification: not reported.

	n	Average score	Scoring system
Kapoor et al. [27]	16	79.8	MMAS
Okcu and Aktuglu [15]	24	86.6	MMAS
Imren et al. [30]	20	79.6	AOFAS
Rayan et al. [33]	20	86.7	AOFAS
Patra et al. [31]	21	76.3	AOFAS
Sahin et al. [32]	14	80.4	AOFAS

Eight articles reported radiological outcomes. Ovadia et al. described post-operative articular reduction as a critical factor in outcomes following surgical management of TPF [36]. Teeny et al. later adapted the Ovadia & Beal radiological criteria. The Teeny & Wiss criteria were reported by 4/16 papers as radiological scores of reduction adequacy (Table 5). Of 127 patients reported, 63% (n = 80) were deemed to be good-to-anatomic, 30% (n = 38) fair, and 7% (n = 8) poor. The author acknowledges that these criteria are not directly comparable. However, they act as an indicator of radiological reduction post-operatively.

**Table 5:** Radiological outcome scores.

		Teeny & Wiss criteria			
	cohort size, n	Anatomic	Good	Fair	Poor
Osman et al. [29]	30	5	15	6	4
Lovisetti et al. [25]	30	5	23	2	0
Kholeif et al. [26]	15	1	9	4	1
Kapukaya et al. [22]	14	4	6	2	2
		Articular gap, mm			
		Good, <2 mm		Fair, 2-4 mm	Poor, >4 mm
Kapoor et al. [27]	17	5		10	1
Patra et al. [31]	21	7		14	0

Quality and bias analyses

The mean quality score of comparative studies, assessed using the MINORS criteria, was 16.8 ± 1.2 (Table 6).

**Table 6:** Quality scores of comparative studies. MINORS = Methodological Index for Non-Randomized Studies

MINORS tool					
	Okcu and Aktuglu 2004 [15]	Harris et al., 2006 [16]	Bacon et al., 2008 [24]	Imren et al., 2017 [30]	Patra et al., 2017 [31]
Clearly stated aim	2	2	2	2	2
Inclusion of consecutive patients	2	2	2	2	2
prospective collection of data	0	0	0	0	0
End-points appropriate to the aim of the study	2	2	2	2	2
Unbiased assessment of the study endpoint	0	0	0	0	0
Follow-up period appropriate to the aim of the study	2	2	2	2	2
Loss to follow-up <5%	1	2	0	2	1
Prospective calculation of study size	0	0	2	0	0
Adequate control group	2	2	2	2	2
Contemporary groups	2	2	2	2	2
Baseline equivalence of groups	2	0	2	2	0
Adequate statistical analysis	2	2	2	2	2
Total	17	16	18	18	15

Rayan et al. performed the only prospective randomised trial in this systematic review and was judged as low risk of bias, according to the ROB 2 tool (Table 7). The remaining studies were deemed at moderate risk of bias, according to the ROBINS-I tool (Table 8).

**Table 7:** Risk of bias for randomised study. ROB-2 = Risk of Bias 2

ROB-2 tool	
	Rayan et al., 2018 [33]
Risk of bias arising from the randomisation process	Low
Risk of bias due to deviations from the intended intervention	Low
Missing outcome data	Low
Risk of bias in the measurement of outcome	Low
Risk of bias in the selection of reported results	Moderate
Overal risk of bias	Low

**Table 8:** Risk of bias for non-randomised trials. ROBINS-I = Non-Randomised Studies - of Interventions

ROBINS-I					
	Okcu and Aktuglu 2004 [15]	Harris et al., 2006 [24]	Bacon et al., 2008 [24]	Imren et al., 2017 [30]	Patra et al., 2017 [31]
Bias due to confounding	Moderate	Moderate	Moderate	Moderate	Moderate
Bias in selection of participants into the study	Serious	Serious	Serious	Serious	Serious
Bias in classification of interventions	Moderate	Moderate	Moderate	Moderate	Serious
Bias due to deviation from intended interventions	Moderate	Moderate	Moderate	Moderate	Moderate
Bias due to missing data	Serious	Low	Moderate	Low	Moderate
Bias in measurement of outcomes	Moderate	Moderate	Moderate	Moderate	Moderate
Bias in selection of the reported outcomes	Low	Low	Moderate	Moderate	Moderate
Overall judgement of bias	Moderate	Moderate	Moderate	Moderate	Moderate

Discussion

To date, this systematic review is the only one to report complications and radiological and functional outcome measures regarding circular external fixators used to definitively manage TPFs. This systematic review included CEF data from comparative studies in which CEF was compared to alternative modalities of fixation. The potential advantages of CEF in the treatment of TPF have been well reported.

Theoretically, CEF is biomechanically advantageous as it creates a construct in which forces are centred around the long-axis of the bone, therefore minimising cantilever bending [37]. The use of multiple wires in different trajectories in the axial plane allows multi-planar fixation, providing improved resistance to shear and torsional forces. Additionally, tensioned-wire CEF can allow early weight-bearing, causing axial-micromotion, which may encourage bone union [38-43].

Another theoretical advantage of CEF over ORIF is the limitation of secondary insult to the already injured soft tissue envelope. Previously, the use of internal fixation had been thought to be associated with higher rates of infection, resulting in increased use of external fixators either temporarily or definitively [44,45]. Interestingly, the recent meta-analysis by Malik-Tabassum et al. found that deep infection rates were not significantly different between CEF and ORIF. They noted a significantly increased risk of superficial wound infection, attributed to simple pin-site infections, as echoed in this systematic review [46].

This systematic review identified 16 articles, of which 15/16 were level III/IV evidence. Only one was of evidence level II study (Rayan et al. 2018 - prospective randomised study CEF vs. ORIF). The studies were heterogenous in design and participants. The sample sizes were relatively small, with 8/15 reporting fewer than 20 CEF patients.

The proportion of open fractures was variable, with an average of 23.1%. Four articles reported no open fractures. Sahin et al. reported exclusively on open fractures of AO classification 43-C3. Open fractures of increasing severity have been associated with increased infective complications [47]. The requirement for plastic surgical intervention was not analysed.

Detailed patient demographics and comorbidities were not available. It is well recognised that patient-related factors, including diabetes mellitus, peripheral vascular disease and smoking status, significantly affect the post-operative complication and outcomes of patients sustaining fractures around the ankle [48-50]. This could not be addressed in this systematic review.

The severity of the soft tissue and bony injury, resource availability, and surgeon experience are all important factors when deciding to use CEF. Watson et al. performed a prospective study in which lower-severity soft tissue injury TPFs (Tscherne classification 0 or I) underwent ORIF and higher-severity (Tscherne classification II or III and open fractures) underwent CEF. The inference is that CEF is reserved for cases with a poorer soft tissue envelope, therefore, a significant confounder when comparing CEF to alternative modalities. This systematic review, including open and closed fractures, showed that the mean deep infection rate in CEF was 5.0%, and no amputations were reported.

The mean follow-up time was 35.3 ± 13.4 months. The typical onset of symptomatic post-traumatic osteoarthritis, with radiological and/or clinical features, occurs within two to four years [51,52]. Therefore, the follow-up time for this systematic review is reasonable, but some late presentations may have been missed. The rate of secondary procedures or amputation as a result of the development of post-traumatic arthritis beyond the follow-up period is unknown.

There was heterogeneous reporting of functional outcomes. Only six articles reported stratified functional outcome scores (see Table 4) [53]. The AOFAS includes objective and subjective domains including pain, function and alignment. The AOFAS is commonly reported numerically and not stratified. Though similar tools, they are not directly comparable. The AOFAS and Mazur scores both remain unvalidated. Ceccarelli et al. reported poor correlation between AOFAS scores and Medical Outcomes Study SF-36 (short-form, 36-item questionnaire) with regard to Achilles tendon repair [54]. SooHoo et al. found poor correlation between AOFAS and SF-36 for elective foot and ankle surgery [55]. Conversely, Ibrahim et al. report moderate correlation and satisfactory reliability between pre- and post-operative AOFAS and SF-36, and concluded that it has acceptable validity [56]. This systematic review showed that, according to PROMS (including MMAS and AOFAS), approximately one-third achieve excellent, one-third good, and one-third fair or poor outcomes (see Table 4). Despite the debatable validity of these scoring tools, these results are in keeping with the literature regarding TPF outcomes. Moreover, this provides pertinent information as to the overall outcomes while counselling patients in the perioperative and postoperative setting and gaining informed consent.

Limitations

This systematic review identified 16 studies, the majority of which were retrospective case or cohort studies, of level III or IV evidence. They were of moderate quality and had a moderate risk of bias. Only one prospective randomised trial was identified. CEF is not routinely practiced in all institutions; therefore, there may be inherent bias through lack of availability. Each study was relatively small, with an average of 19 patients per study. Reporting of demographic and outcome data was heterogenous, as was the use of classification and outcome scoring tools.

## Conclusions

This systematic review is the first to report the clinical and functional outcomes of TPFs treated definitively with CEF. It found a mean frame time of 4.5 months and union time of 5.5 months, highlighting the importance of educating patients regarding the duration of treatment during the consent process. Additionally, 1-in-30 underwent non-union and 1-in-10 mal-union. Around 3% required arthrodesis in the medium term. Only one in three achieved an excellent functional outcome, and approximately 10% had a poor functional outcome. While large, randomised, and prospective studies are lacking, this systematic review provides a valuable collation of evidence for surgeons and patients undergoing CEF for the management of these complex injuries.
